# High Molecular Weight Polyproline as a Potential Biosourced Ice Growth Inhibitor: Synthesis, Ice Recrystallization Inhibition, and Specific Ice Face Binding

**DOI:** 10.1021/acs.biomac.2c01487

**Published:** 2023-02-21

**Authors:** Nicola Judge, Panagiotis G. Georgiou, Akalabya Bissoyi, Ashfaq Ahmad, Andreas Heise, Matthew I. Gibson

**Affiliations:** † Department of Chemistry, RCSI University of Medicine and Health Sciences, Dublin 2, Ireland; ‡ Department of Chemistry, University of Warwick, Gibbet Hill Road, CV4 7AL Coventry, U.K.; § Division of Biomedical Sciences, Warwick Medical School, University of Warwick, Gibbet Hill Road, CV4 7AL Coventry, U.K.; ∥ Science Foundation Ireland (SFI) Centre for Research in Medical Devices (CURAM), RCSI, Dublin 2, Ireland; ⊥ AMBER, The SFI Advanced Materials and Bioengineering Research Centre, RCSI, Dublin D02, Ireland

## Abstract

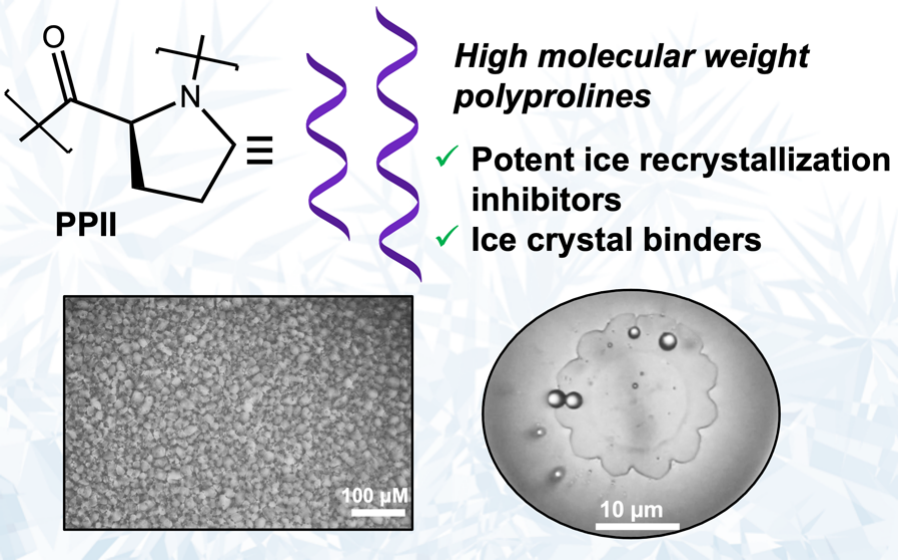

Ice-binding proteins (IBPs) from extremophile organisms can modulate ice formation and growth. There are many (bio)technological applications of IBPs, from cryopreservation to mitigating freeze–thaw damage in concrete to frozen food texture modifiers. Extraction or expression of IBPs can be challenging to scale up, and hence polymeric biomimetics have emerged. It is, however, desirable to use biosourced monomers and heteroatom-containing backbones in polymers for *in vivo* or environmental applications to allow degradation. Here we investigate high molecular weight polyproline as an ice recrystallization inhibitor (IRI). Low molecular weight polyproline is known to be a weak IRI. Its activity is hypothesized to be due to the unique PPI helix it adopts, but it has not been thoroughly investigated. Here an open-to-air aqueous *N*-carboxyanhydride polymerization is employed to obtain polyproline with molecular weights of up to 50000 g mol^–1^. These polymers were found to have IRI activity down to 5 mg mL^–1^, unlike a control peptide of polysarcosine, which did not inhibit all ice growth at up to 40 mg mL^–1^. The polyprolines exhibited lower critical solution temperature behavior and assembly/aggregation observed at room temperature, which may contribute to its activity. Single ice crystal assays with polyproline led to faceting, consistent with specific ice-face binding. This work shows that non-vinyl-based polymers can be designed to inhibit ice recrystallization and may offer a more sustainable or environmentally acceptable, while synthetically scalable, route to large-scale applications.

## Introduction

Ice-binding proteins are produced by many species to control the formation and growth of ice crystals.^1,2^ The ability to recognize ice in a vast excess of water is a remarkably challenging biological recognition achievement, and the fundamental mechanisms of how this is achieved are under active investigation.^3−5^ The potential to control ice formation and growth has many applications across frozen food,^6,7^ to mitigate freeze/thaw damage in concrete,^8^ or to prevent ice formation on aircraft wings or turbine blades.^9,10^ Ice-binding proteins have been found to mitigate cell stress during cryopreservation due to their ice recrystallization inhibition (IRI) activity.^11−13^ For all these applications, a key challenge has been reproducing the complex function of ice-binding proteins using synthetic platforms that benefit from scalability and tunability, which may not be possible with extracted or recombinant proteins.^14^ Polymeric mimics have emerged, with poly(vinyl alcohol), PVA, being the most studied.^15−20^ PVA functions by a different molecular level mechanism than IBPs, with hydrogen bonding from the hydroxyls to the ice face being essential.^21,22^ It is important to note that polyhydroxylation is not a predictor of IRI activity, with most hydroxylated polymers showing no significant activity.^23,24^ Polymers and self-assembled structures with amphipathic structures have been reported to show IRI activity^25,26^ as have nanocellulose,^27^ self-assembling peptides,^28^ and some nanoparticles.^29^ Ben et al. have pioneered the use of small molecules, such as alkyl glycosides which can limit ice recrystallization^30−32^ and have been shown to enhance red blood cell cryopreservation.^33^

In the design of polymer mimics for future *in vivo* applications or deployment in the environment, their fate must be considered to prevent the accumulation of nondegradable materials.^34^ Ester linkages have been incorporated in poly(vinyl alcohol) and polyampholytes through radical ring-opening polymerization^35,36^ but degrade to shorter polymers rather than to small molecules. A strategy to use biosourced building blocks to obtain materials that are potentially hydrolyzable or enzymatically cleavable (depending on the sequence) is to use poly(amino acid)s derived from *N*-carboxyanhydride, NCA, polymerization.^37^ NCAs are typically formed by the phosgenation of natural or synthetic amino acids, which can undergo ring-opening polymerization (ROP) to yield poly(amino acid)s. By choice of initiator, the polymerization can be controlled, allowing for an extensive range of molecular weights, dispersities and architectures to be synthesized and implemented into biomedical applications.^38−40^ Poly(amino acid) materials offer an appealing solution to degradable and biosourced materials to replace oil-derived monomers.^41^ For instance, Wooley and co-workers showed polypeptide-based, recyclable, redox batteries.^40^

The relatively simple primary amino acid sequence of antifreeze glycoproteins^42,43^ (alanine–alanine–threonine) suggests (to a first approximation) that simple homo-/copolypeptides with IRI activity can be discovered. Knight et al.,^44^ and later Cameron and co-workers,^45^ reported that poly(hydroxyproline) had IRI activity. This is an exciting target, as the antifreeze glycoproteins (for which a crystal structure does not exist) show a secondary structure similar to a polyproline II (PPII) helix.^46,47^ This also suggests that the PPII is a valuable design target, although several PPII designed peptides failed to show IRI activity.^48^ In 2017, Gibson and co-workers explored how short polyprolines,^49^ rather than poly(hydroxyprolines) (which when bought commercially can actually contain significant polyproline content), could lead to activity, supporting the emerging hypothesis that hydrophobic faces (rather than hydroxyls) engage ice in many IBPs.^50^ Although the oligoprolines were relatively weak in terms of IRI (not inhibiting all ice growth even at 20 mg mL^–1^), they were found useful for cryopreservation.^49,51^ It is important to note that this is distinct from the effect of the amino acid l-proline in cryopreservation as a protective osmolyte.^52,53^ Alanine/lysine copolymers have also been reported to show IRI activity;^54,55^ for biomedical applications, the net cationic charge may lead to cytotoxicity. Poly(serine) has been tested for IRI,^24^ but the magnitude reported was essentially identical with a negative control of PEG.^23^ High molecular weight polyproline has not been explored for IRI as it is challenging to obtain by NCA polymerizations due to proline NCA’s unique bicyclic structure which typically leads to slow polymerization kinetics and relatively low molecular weight materials until copolyemerized.^56,57^ Recent advances in proline NCA polymerizations now make high molecular weight polyproline accessible.^58^ Lu and co-workers have reported the water-assisted controlled ROP of ProNCA in ACN/water mixture affording well-defined polyproline II helices.^59^

Considering the above, this work explores the synthesis and ice-binding protein-mimetic activity of polyproline as a step toward identifying biosourced and scalable ice recrystallization inhibitors. Polyproline was synthesized by aqueous open-air ring-opening *N*-carboxyanhydride polymerization, giving rise to polypeptides of up to 50 kg mol^–1^. The peptides were found to be potent ice recrystallization inhibitors, with the highest molecular weight peptides inhibiting all growth below 5 mg mL^–1^. The peptides were found to specifically bind ice crystals, as shown by cryomicroscopy.

## Materials and Methods

### Materials

All chemicals were used as supplied unless otherwise stated. Boc-proline, Boc-hydroxyproline, and triphosgene were purchased from Fluorochem. Hexylamine, epichlorohydrin, and acetonitrile were purchased from Sigma-Aldrich. Formvar-carbon-coated (300 mesh) copper grids were purchased from EM Resolutions. Ultrapure water used for buffers was Milli-Q grade (18.2 mΩ resistance).

### Characterization Techniques

#### NMR Spectroscopy

^1^H NMR and ^13^C NMR spectra were recorded at 400 MHz on a Bruker Advance spectrometer, with chloroform-*d* (CDCl_3_), DMSO-*d*_6_ ((CD_3_)_2_SO), and D_2_O as the solvent. Chemical shifts of protons are reported as δ in parts per million (ppm) and are relative to tetramethylsilane (TMS) at δ = 0 ppm when using CDCl_3_ or solvent residual peak (H_2_O δ = 4.79 ppm/DMSO δ = 2.50 ppm). ^1^H-DOSY was recorded on the previously described spectrometer, and diffusion coefficients are reported in cm^2^ s^–1^, obtained using the MestReNova 6.02 software Bayesian method. Diffusion coefficients were calculated from the intensity of the pyrrole (∼3.8 ppm) signal of the l-Pro side chain using a monoexponential decay equation, which is a simplified version of the Stejskal–Tanner function.

#### Size Exclusion Chromatography (SEC)

SEC analysis of polyproline (PPro) homopolymers was performed on an Agilent Technologies Infinity 1260 MDS instrument equipped with differential refractive index (DRI), light scattering (LS), and viscometry (VS) detectors. The column set used was a PL Aquagel-OH MIXED-M column. The mobile phase used was a H_2_O:ACN mixture (80:20) + 0.1 M NaNO_3_. Column oven and detector temperatures were regulated to 40 °C, at flow rate 1 mL/min. Poly(ethylene oxide) standards (Agilent EasyVials) were used for calibration between 100 and 500000 g mol^–1^. Analyte samples were filtered through a hydrophilic GVWP membrane with 0.22 μm pore size before injection. Number-average molecular weights (*M*_n_), weight-average molecular weights (*M*_w_), and dispersities (*Đ*_M_ = *M*_w_/*M*_n_) were determined by conventional calibration and universal calibration using Agilent GPC/SEC software.

SEC analysis of polysarcosine homopolymers was performed using a PSS SECurity GPC system equipped with a PFG 7 μm 8 × 50 mm precolumn, a PSS 100 Å, 7 μm 8 × 300 mm column, and a PSS 1000 Å, 7 μm 8 × 300 mm column in series and a differential refractive index (RI) detector at a flow rate of 1.0 mL min^–1^ in 1,1,1,3,3,3-hexafluoro-2-propanol (HFiP). The system was calibrated against Agilent Easi-Vial linear poly(methyl methacrylate) (PMMA) standards and analyzed by PSS winGPCUniChrom. All SEC samples were prepared using a concentration of 2 mg mL^–1^ and were filtered through a 0.2 μm Millipore filter prior to injection.

#### Fourier Transform-Infrared (FTIR) Spectroscopy

FTIR spectroscopy measurements were performed using an A PerkinElmer Spectrum 100 spectrometer in the range 650–4000 cm^–1^ and analyzed using OMNIC software.

#### Dynamic Light Scattering (DLS)

Hydrodynamic diameters (*D*_h_) and size distributions of PPro polymer samples were determined by DLS using a Malvern Zetasizer Nano ZS with a 4 mW He–Ne 633 nm laser module operating at 25 °C. Measurements were performed at an angle of 173° (backscattering), and results were analyzed using Malvern DTS 7.03 software. All determinations were repeated 5 times with at least 10 measurements recorded for each run.

#### Splat Ice Recrystallization Inhibition Assay

Splat cooling assays were performed as previously described.^16,60^ Briefly, a 10 μL sample was dropped 1.40 m onto a chilled glass coverslip, resting on a thin aluminum block cooled to −78 °C placed on dry ice. Upon hitting the coverslip, a wafer with diameter of approximately 10 mm and thickness 10 μm was formed instantaneously. The glass coverslip was transferred onto the Linkam cryostage and held at −8 °C using liquid nitrogen for 30 min. Photographs were obtained using an Olympus CX 41 microscope with a UIS-2 20*x*/0.45/∞/0-2/FN22 lens and crossed polarizers (Olympus Ltd.), equipped with a Canon DSLR 500D digital camera. Images were taken of the initial wafer (to ensure that a polycrystalline sample had been obtained) and again after 30 min. Image processing was conducted using ImageJ. In brief, the number of ice crystals in the field of view was measured for each photograph. The average (mean) of these three measurements was then calculated to find the mean grain area (MGS). The average value and error were compared to that of PBS solution, as appropriate, as a negative control.

### Synthesis of Proline *N*-Carboxyanhydride

Based on previous method.^59^ Boc-l-proline (5 g, 23.2 mmol) and epichlrohydrin (8.6 g, 92.9 mmol) were dissolved in acetonitrile (50 mL) at 0 °C, and triphosgene (3.45 g, 11.6 mmol) was added in one portion. The reaction proceeded open to air at 0 °C for 2.5 h until no solids remained; the solution was filtered and reduced *in vacuo* at 40 °C for 30 min. The flask of the remaining solution was filled with N_2(g)_ using a cannula, then precipitated into a large excess of hexane, and stored overnight at −18 °C. The resulting solution was reduced to 1/3rd volume *in vacuo* under vigorous stirring. The crude NCA oil was dissolved in ethyl acetate and reprecipitated into hexane twice to afford a solid. The NCA was dried overnight over P_2_O_5_ to afford a white powder (2.4 g, yield 85%). ^1^H NMR (400 MHz, CDCl_3_, 293 K) δ: 4.33 (m, 1H), 3.79 (dt, *J* = 11.4, 7.6 Hz, 1H), 3.33 (ddd, *J* = 11.5, 8.5, 4.9 Hz, 1H), 2.32 (m, 1H), 2.17 (m, 2H), 1.95 (m, 1H). ^13^C NMR (101 MHz, CDCl_3_) δ: 168.9, 155.0, 63.17, 56.7, 27.26, and 27.0

### Synthesis of Polyproline

Proline NCA was dissolved in an acetonitrile/H_2_O (1:1 v/v) at a concentration of 50 mg mL^–1^ in a small vial open to air. Hexylamine was added from a stock solution acetonitrile/H_2_O (1:1 v/v) directly in one portion, and the reaction proceeded as gas bubbles were evident. Once the NCA peaks at 1823 and 1764 cm^–1^ had disappeared, the reaction was quenched by addition of excess of H_2_O. The solution was dialyzed (MWCO = 3.5 kDa) for 3 days and lyophilized.

#### 20K and 50K Targeted *M*_w_

The previous procedure was adapted so that NCA proline was added portionwise in 2 or 5 doses for the 20K and 50K targeted *M*_w_, respectively. ^1^H NMR (400 MHz, D_2_O, 293 K) δ: 4.70 (1H), 3.68 (2H), 2.30 (1H), 2.03–1.87 (3H). ^13^C NMR (101 MHz, D_2_O) δ: 171.6, 58.51, 47.55, 27.86, 24.50.

## Results and Discussion

**Figure 1:**
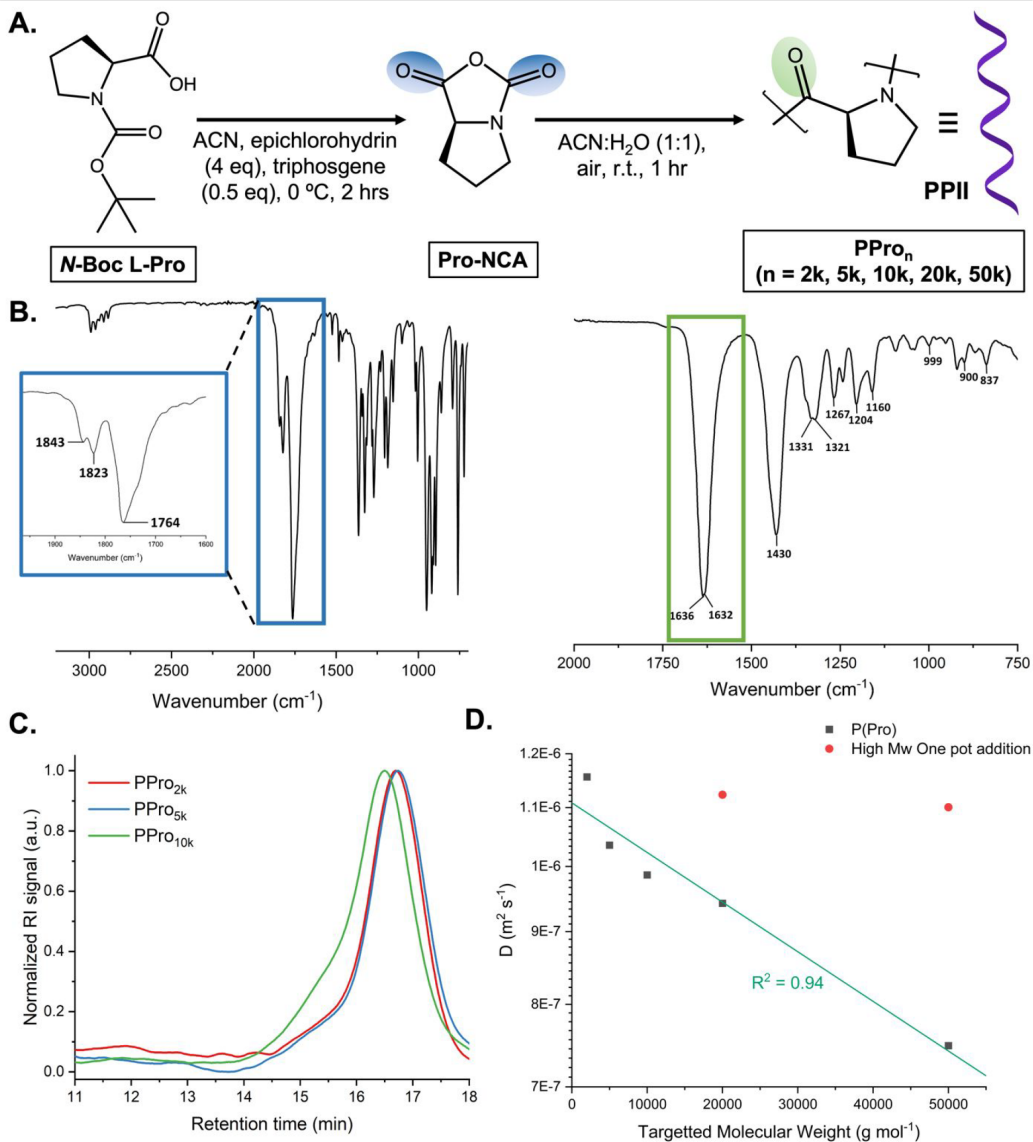
(A) Schematic of the synthetic route followed for the synthesis of PPro_*n*_ (*n* = 20, 52, 103, 206, 515). (B) FTIR of Pro-NCA (left) (highlighting asymmetric carbonyl stretch at 1840 and 1760 cm^–1^) and PPro_20_ (right) (highlighting carbonyl stretch of polymer backbone). (C) Normalized SEC-RI distributions of PPro_*n*_ (*n* = 2k, 5k, 10k) in H_2_O:ACN (8:2) + 0.1 M NaNO_3_. (D) Diffusion coefficients determined by DOSY for PPro_*n*_ (*n* = 2k, 5k, 10k, 20k, 50k) as a function of targeted molecular weight.

**Figure 2:**
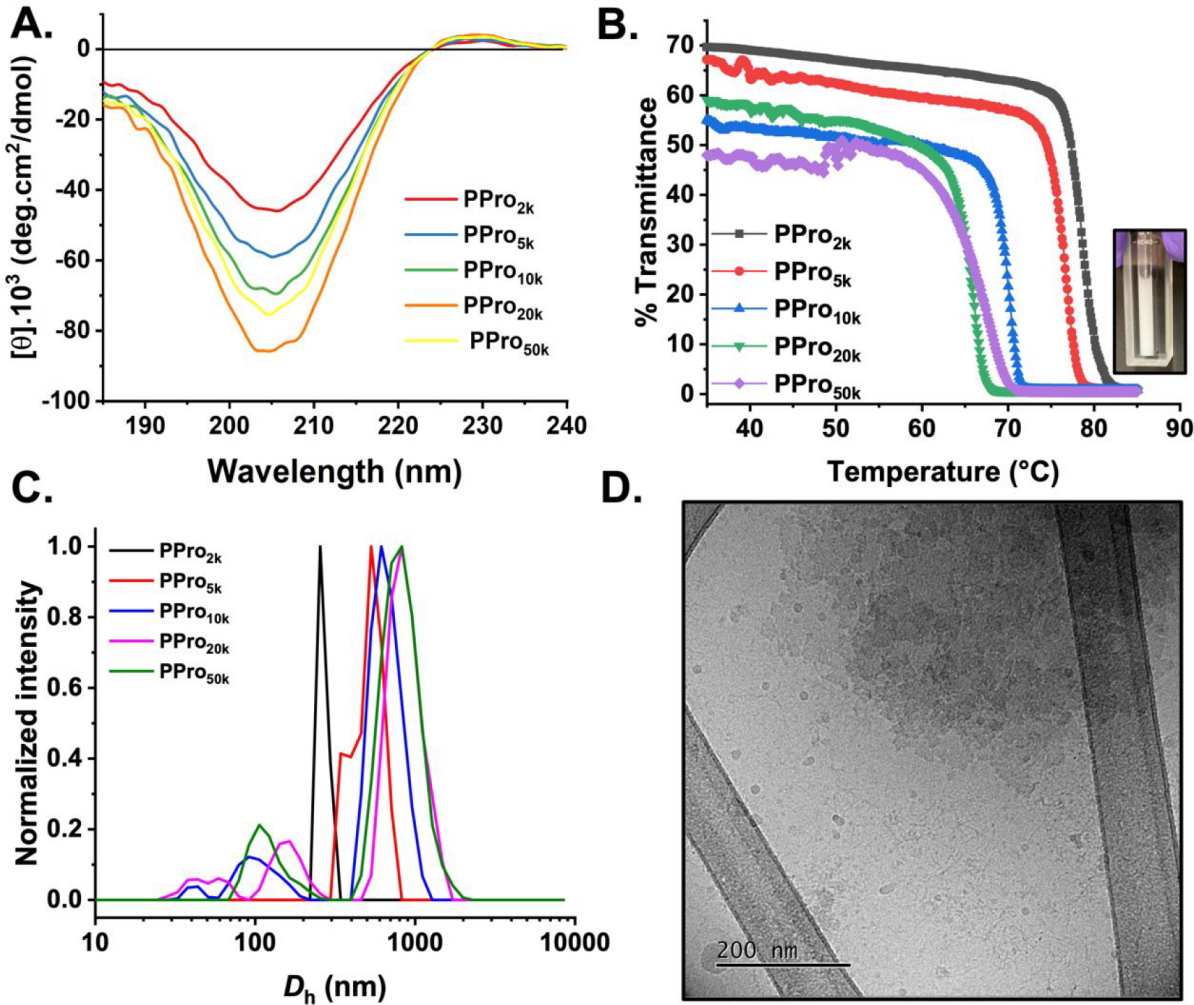
Solution properties of polyproline. (A) CD spectra of PPro of varied DP (1 mg mL^–1^ in PBS media). (B) Turbidimetry curves of PPro (5 mg mL^–1^ in PBS). (C) Intensity-weighted size distributions of PPro obtained from DLS. (D) Representative cryo-TEM image of PPro_50k_.

Using the improved one step procedure reported by Tian et al.,^59^ proline NCA was synthesized from *N*-Boc l-proline using triphosgene and epichlorohydrin as an HCl scavenger (Figure 1). The NCA was fully characterized by ^1^H, ^13^C, COSY-NMR, and FTIR, confirming the asymmetric carbonyl stretch (1840 and 1760 cm^–1^) which is characteristic of NCA formation. The lack of FTIR peaks at 1640 and 1630 cm^–1^ indicates that the absence of polymeric side reactions and residual starting material, respectively. Full characterization is included in the Supporting Information (Figures S1–S4). One drawback of using NCA monomers to obtain high-*M*_w_ polypeptides is their moisture sensitivity, thereby requiring stringent conditions to achieve a controlled polymerization. Recently, the ability to suppress or outpace the hydrolysis by tuning the environment has been reported. Song et al. utilized a biphasic DCM/aqueous system which was shown to produce high molecular weight polypeptides with narrow distributions.^61^ Alongside this the development of ROPISA has allowed for NCA monomers to polymerize within a basic aqueous environment to outpace the hydrolysis.^62^ With regard to NCA-proline the methodology reported by Hu et al. was remarkable, using DFT calculations it was shown that the presence of water was promoting polymerization, and hence this method is employed here.^58^ In this method acetonitrile/water mixtures are used which leads to fast kinetics and high yields and prevents precipitation of growing polyproline chains (Figure 1A). It should be noted this is nonobvious due to the well-known susceptibility of NCA’s to hydrolysis,^56^ but the rapid polymerization outcompetes this side reaction. Despite the fast polymerization kinetics, it was found that when targeting molecular weights above 10000 g mol^–1^, using hexylamine as the initiator, the monomer had to be added in batches to avoid hydrolysis caused by prolonged exposure to water. The polypeptides produced were characterized by SEC; however, the highest molecular weights targeted 20000 and 50000 g mol^–1^ were unable to be analyzed by SEC due to solubility issues and the rigidity of the structure (Table 1). The 2000, 5000, and 10000 g mol^–1^ molecular-weight-targeted polypeptides showed monomodal traces with the expected retention time shifts, but the *M*_n_ values obtained were considered unreliable due to the rigidity of the structure, solubility, and the differences between the PPII helix and PEG standards. Therefore, DOSY ^1^H NMR spectra in D_2_O were used to confirm the range of molecular weights targeted was achieved as the diffusion coefficients shifts should be universal for a given polymer independent of standards.^63^ The molecular weight targeted was plotted against diffusion coefficient measured for the polyprolines which gave an *R*^2^ value 0.94 for the linear fit. It was hence concluded that the molecular weights targeted had been achieved as a result of the linear nature of the diffusion coefficients obtained. The novel polymerization reaction media allows for the direct synthesis of the water-soluble PPII helix as opposed to the organic solvent soluble PPI helix of previous reports.^56,57,64^ This was confirmed by FTIR as peaks at 1204 and 1160 cm^–1^ are of the same relative intensity and peaks at 1360 and 960 cm^–1^ are absent. Alongside this the CD spectra displayed a typical trace of a left-handed PPII helix (Figure 2A). Therefore, it was concluded that five different molecular weight polyprolines ranging from 2000 to 50000 g mol^–1^ displaying the desired PPII helix were successfully synthesized.

**Table 1:** Poly(l-proline)s Synthesized

polymer	[M]:[I]^a^	monomer batches^b^	*M*_n,theo_ (g mol^–1^)	*M*_n,SEC_ (g mol^–1^)^c^	*Đ*_M_^c^	*D*^d^ (m^2^ s^–1^)
polyPro_2k_	20	1	2000	2700	1.38	1.15 × 10^–6^
polyPro_5k_	52	1	5000	3100	1.57	1.03 × 10^–6^
polyPro_10k_	103	1	10000	3600	1.52	9.86 × 10^–7^
polyPro_20k-1_	206	1	20000			1.12 × 10^–6^
polyPro_20k_	206	2	20000			9.42 × 10^–7^
polyPro_50k-1_	515	1	50000			1.10 × 10^–6^
polyPro_50k_	515	5	50000			7.48 × 10^–7^

a Ratio achieved after all monomer additions.

b Mass of monomer added was divided evenly between additions.

c *M*_n_ and *Đ*_M_ values calculated from PEG standards using H_2_O:ACN (80:20) as the eluent.

d Diffusion coefficients obtained from DOSY ^1^H NMR.

Polymers featuring lactams (cyclic amides) are known to display thermoresponsive LCST (lower critical solution temperature) behavior in aqueous solution,^65−67^ and hence these polymers were evaluated for their cloud point (noting that the LCST is the minima of the composition–temperature curve^68^) at 1 mg mL^–1^ in phosphate buffered saline (PBS) (Figure 2B). The lowest molecular weight polyproline showed a cloud point of 80 °C, with the cloud point decreasing as molecular weight increased to ∼65 °C for the 50 kg mol^–1^ polymers. This *M*_w_ dependence on cloud point is commonly observed for other thermoresponsive polymers.^69,70^ During these experiments, it was observed that concentrated solutions were cloudy (hence in Figure 2B transmittance does not start at 100%), suggesting some intrinsic aggregation or assembly. Dynamic light scattering (DLS) confirmed aggregation was occurring, which was further validated by cryogenic and dry-state TEM (transmission electron microscopy) (Figures 2C,D and S9). While dry-state TEM imaging showed the presence of needle-shaped fibers, cryo-TEM suggests the presence of aggregates solely, and hence the dry-state TEM could be a drying effect rather than representative of the solution state. This aggregation does not prevent their later application, but is important to consider when measuring any properties that assemblies are present, not just freely dissolved individual chains. In fact, for the later IRI testing, hydrophobicity is known to be crucial, and hence this limit of solubility may be useful, as seen for facially amphiphilic glycopolymers.^26^ It was recently demonstrated that polyproline shows large hysteresis in its LCST transitions matching the observations found here.^71^

**Figure 3:**
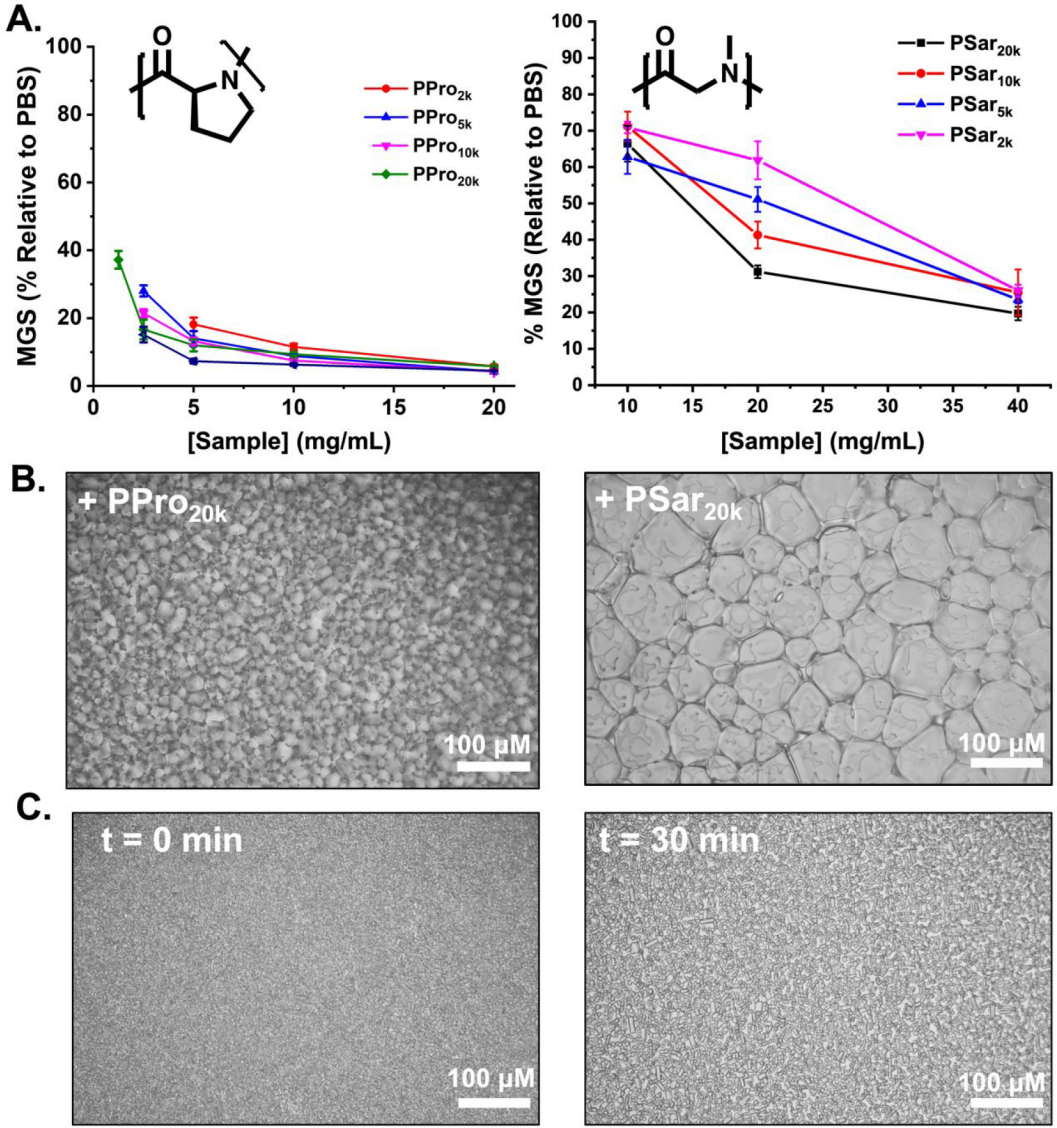
IRI activity of polypeptides. (A) Dose-dependent IRI activity of poly(l-proline) and poly(sarcosine). (B) Example cryomicrographs from the “splat” assay after 30 min annealing at −8 °C in PBS for 10 mg mL^–1^ of PPro_20k_ (left) and PSar_20k_ (right). (C) Example cryomicrographs in a sucrose sandwich (45 wt % sucrose) assay at −20 °C containing 10 mg mL^–1^ of PPro_50k_.

A previous report on oligoprolines (*M*_w_ < 10 kg mol^–1^) reported weak ice recrystallization inhibition (IRI) activity, limiting growth to 50% MGS at 20 mg mL^–1^.^23,49^ It was hypothesized that the amphiphilic PPII backbone was a crucial feature, but this has not been widely explored. Other IRI active polymers typically show increased activity as molecular weight increases,^14,21,54^ and hence it was important to explore the IRI of the present polyproline library to determine if simply increasing the molecular weight would lead to enhanced properties. To assess IRI, the splat assay was used. This assay allows separation of nucleation and growth effects by seeding a polycrystalline ice wafer which is then monitored for growth while held at subzero temperatures.^16,44^ In the absence of IRI activity the crystals grow, but if an IRI active agent is present, the ice growth (recrystallization) is suppressed. The data are reported as mean grain size (MGS) relative to a negative of the buffer alone. It is crucial to note that saline (or other additives) is essential to form a eutectic phase to prevent false positives, and hence phosphate buffered saline was used ([NaCl] = 0.137 M).^23^Figure 3A shows a full analysis of the dose-dependent IRI of the library of polyprolines. All of them were capable of inhibiting ice growth and were more active than previous reports. However, the materials used here were obtained by a controlled polymerization method, but previous reports were supplier-provided molecular weights containing large fractions of low molecular weight material explaining this deviation.^49^ The higher molecular weights were more active inhibiting growth below 5 mg mL^–1^, placing them in the medium activity range of antifreeze protein mimetics (less active than PVA or antifreeze proteins).^23^ It is important to also note that due to the propensity for the polyprolines to aggregate, the similar activity seen across the panel could be due to larger aggregates dominating the observed activity. Figure 3B shows IRI activity for a control polypeptide of polysarcosine (also obtained by NCA polymerization; see the Supporting Information for the synthetic procedure, Table S1 and Figures S5–S8). For all molecular weights, polysarcosine was less active, and it also highlighted a key feature of IRI testing that any macromolecule of sufficiently high concentration will slow ice growth and hence the importance of negative controls.^23^Figures 3C,D show example cryomicrographs of the polypeptides in ice wafers highlighting the IRI activity. It is important to note that the IRI activity of the polyprolines here is greater than previous reports of short oligoprolines^49,51^ and demonstrates that a simple homopolypeptide can be deployed to prevent ice recrystallization. This may offer advantages compared to all-carbon backbone polymers in the context of making these degradable and from biosourced monomers.

A second assay was also employed to further validate the above IRI observations—the “sucrose sandwich”.^72^ This assay is conducted in concentrated sucrose which reduces the total ice fraction (compared to the splat assay). After 30 min annealing at −20 °C (note: this assay uses lower temperatures than the splat) the control sample containing no additives showed extensive recrystallization (Figure S10), but 10 mg mL^–1^ of PPro_50k_ limited the ice growth. This secondary validation confirms that polyproline has the potential to be a potent IRI agent, so long as the *M*_w_ is sufficiently high.

**Figure 4:**
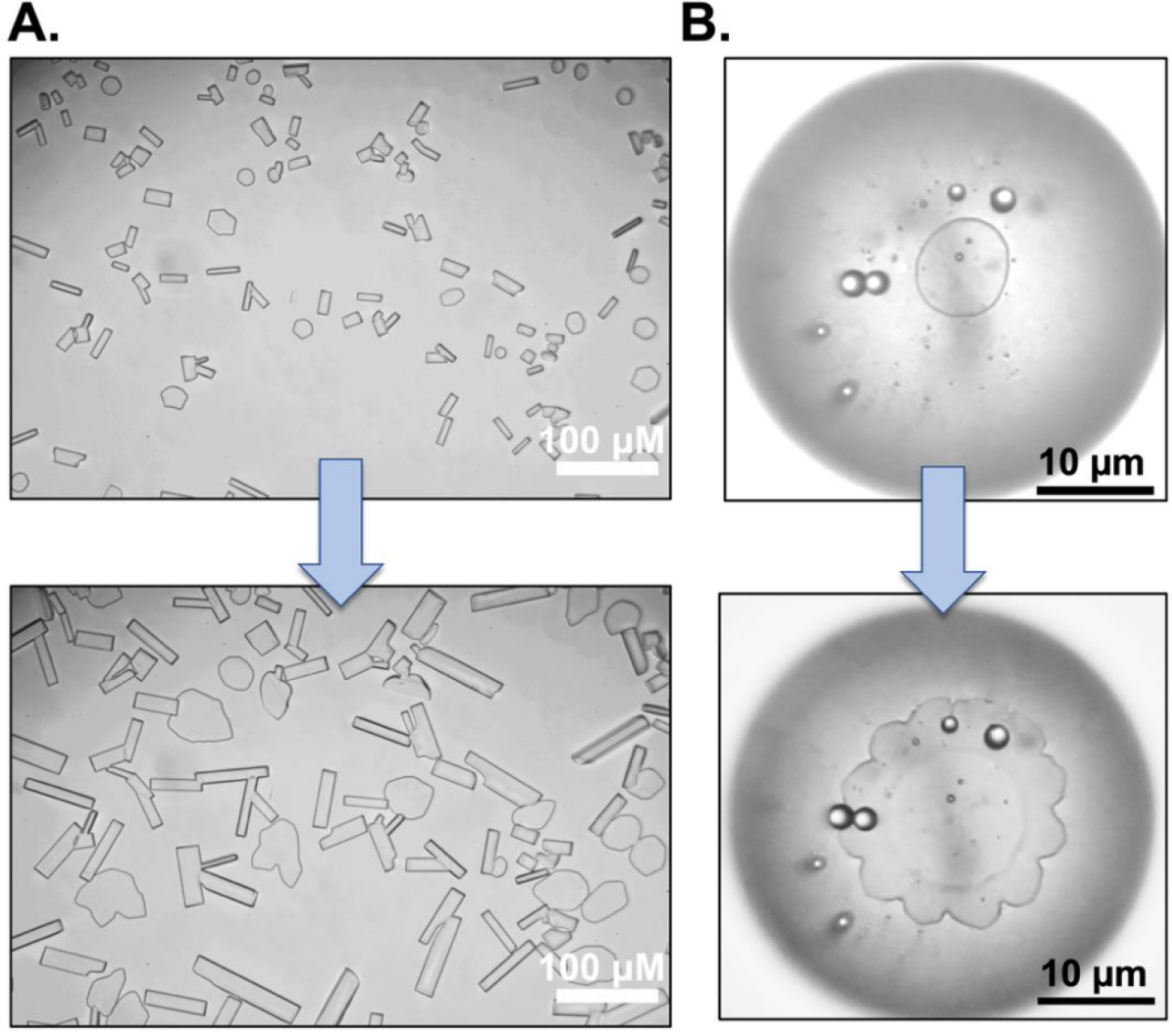
(A) Modified “sucrose sandwich” ice shaping assay images showing rectangular and blunt needle shape for 2 mg mL^–1^ PPro_50k_ in 45 wt % sucrose solution before and after 1 h growth. (B) single crystal ice shaping using 2 mg mL^–1^ PPro_50k_ showing formation of flower-shaped ice crystal as the ice crystal grows.

Ice binding proteins (IBPs) function by binding to specific ice crystal planes, which results in their macroscopic properties of ice recrystallization inhibition, thermal hysteresis, and dynamic ice shaping,^2,73^ as do some synthetic polymers^20,21^ and self-assembled structures.^25^ However, the magnitude of IRI activity varies, separately from the extent of other properties associated with ice binding^23,74^ and some materials can display IRI without any evidence of ice binding.^75^ In short, the macroscopic properties of IRI, thermal hysteresis and dynamic ice shaping^76^ may be induced by different molecular level interactions.^14^Figure 4A shows ice crystals grown in 45% sucrose solution with polyproline, which did not reveal significant evidence of faceting, which would indicate specific ice crystal binding. To further probe this, Figure 4B shows single ice crystal images from a nanoliter osmometer grown in the presence of PPro_50k_. In this assay a single ice crystal is obtained by melting polycrystalline ice until a single crystal remains and then cooled to promote ice growth. In the absence of an ice binder circular crystals are expected, but facets (different shapes) can be seen if there is specific ice face binding. As can be seen, as the ice crystal grows (and before burst growth occurs) faceting can be seen with the formation of pits where there is no ice growth. Similar morphologies are obtained for PVA which binds to the prism planes of ice.^20^ This experiment confirms that polyproline can bind to ice crystals faces. No thermal hysteresis was observed, due to the rapid onset of growth upon cooling. This supports our hypothesis that the PPII helix adopted by polyproline can mimic the PPII-type helix found in antifreeze glycoproteins,^47^ where the hydrophobic face engages the ice face,^50^ which is distinct from e.g. poly(vinyl alcohol) which forms hydrogen bonds to the ice.^21,22^ This is particularly important as it suggests that a simple homopolypeptide sequence is sufficient to bind ice, although the magnitude of effect seen here could be due to the above-discussed aggregates. We note that a 14 amino acid cyclic sequence (more complex sequence, but fewer amino acids) is very potent.^77^

## Conclusions

Here we report the synthesis and application of high molecular weight polyproline as a biomimetic ice recrystallization inhibitor (IRI), demonstrating that potent IRI activity could potentially be introduced into materials derived from renewable sources, as opposed to previously reported polymeric IRIs. Our hypothesis is based on the observations that antifreeze glycoproteins have a polyproline II helix and that the amphipathic structure is a key motif for activity. We have previously reported that oligoprolines showed weak IRI activity limited by the molecular weight. High molecular weight polyprolines were obtained here by aqueous, open to air, *N*-carboxyanhydride (NCA) polymerization, enabling access to polymers with molecular weights as high as 50000 g mol^–1^, which is challenging to achieve with standard NCA polymerization of proline. Because of the rigid nature of the PPII helix, a DOSY NMR method was required to obtain molecular weights of the larger polyprolines, whereas the shorter could be analyzed by conventional size exclusion chromatography. The polyprolines were observed to show molecular weight dependent lower critical solution temperature and potentially assembled at higher concentrations. Ice recrystallization inhibition activity was assessed using both the “splat” and “sucrose sandwich” assays demonstrating polyproline is a potent IRI. The highest *M*_w_ polyproline was able to inhibit all ice growth below 5 mg mL^–1^, which is significantly more active than the previously reported proline oligomers which failed to fully inhibit ice growth even at 20 mg mL^–1^. Single ice crystal shaping assays showed faceting, confirming that polyproline could bind ice crystal faces. These results demonstrate that structural simplification of ice binding proteins to (single) amino acid homopolymers can retain the key IRI activity and even ice-crystal face recognition properties, without a complex primary sequence. These polypeptides are easy to obtain and crucially can be prepared from biosourced starting materials, although it is important to note that a sustainable polymerization method was not explored here, but alternatives such as step-growth or enzymatic polymerization could be employed. The polypeptides also have heteroatoms in their backbone and hence have potential to be hydrolyzed, unlike other IRI active polymers such as poly(vinyl alcohol).

## Data Availability

Any additional research data supporting this publication can be found at http://wrap.warwick.ac.uk.

## Supporting Information Available

Characterization of the monomers and polymers, synthesis of polysarcosine, and additional experimental details (PDF)
