# Supplementary material for: High Molecular Weight Polyproline as a Potential Biosourced Ice Growth Inhibitor: Synthesis, Ice Recrystallization Inhibition, and Specific Ice Face Binding
Source: Biomacromolecules. 2023 Feb 21;24(6):2459–68. doi: 10.1021/acs.biomac.2c01487 (PMC10265654; doi:10.1021/acs.biomac.2c01487)
Supplement: Supplementary file 1 — bm2c01487_si_001.pdf [file bm2c01487_si_001.pdf]

Supporting Information

High Molecular Weight Polyproline as a Potential Bio-Sourced Ice Growth Inhibitor: Synthesis, Ice Recrystallization Inhibition and Specific Ice Face Binding

*Nicola Judge,[1†] Panagiotis G. Georgiou,[2†] Akalabya Bissoyi,[3] Ashfaq Ahmad,[3] Andreas Heise,[1,4,5]*and Matthew I. Gibson[2,3]**

[1] Department of Chemistry, RCSI University of Medicine and Health Sciences, Dublin 2, Ireland.

[2] Department of Chemistry, University of Warwick, Gibbet Hill Road, CV4 7AL, Coventry, UK.

[3] Division of Biomedical Sciences, Warwick Medical School, University of Warwick, Gibbet Hill Road, CV4 7AL, Coventry, UK.

[4] Science Foundation Ireland (SFI) Centre for Research in Medical Devices (CURAM), RCSI, Dublin 2, Ireland.

[5] AMBER, The SFI Advanced Materials and Bioengineering Research Centre, RCSI, Dublin D02, Ireland.

[†] These authors contributed equally to this work

**Corresponding Authors*: andreasheise@rcsi.ie (A.H.), m.i.gibson@warwick.ac.uk (M.I.G.)

Table of Contents

Materials and Methods S2

Materials....................S2

Characterization Techniques....................S2

Experimental Procedures....................S4

Synthetic Procedures S6

Synthesis of NCA Proline....................S7

P (Proline) Synthesis....................S8

Sarcosine NCA Synthesis S10

Polysarcosine (P(L-Sar)) Synthesis S10

References....................S15

Materials and Methods

Materials

All chemicals were used as supplied unless otherwise stated. Boc-Proline, Boc-Hydroxyproline and triphosgene were purchased from Fluorochem. Hexylamine, epichlorohydrin and acetonitrile was purchased from Sigma Aldrich. Formvar-carbon coated (300 mesh) copper grids were purchased from EM Resolutions. Ultra-pure water used for buffers was MilliQ grade (18.2 mΩ resistance).

Characterization techniques

NMR Spectroscopy. ^{1}H-NMR and ^{13}C-NMR spectra were recorded at 400 MHz on a Bruker Advance spectrometer, with chloroform-*d* ($CDCl_3$), DMSO-d_6 ($(CD_3)_2SO$) and D_2O as the solvent. Chemical shifts of protons are reported as δ in parts per million (ppm) and are relative to tetramethyl silane (TMS) at δ = 0 ppm when using $CDCl_3$ or solvent residual peak (H_2O δ = 4.79 ppm/ DMSO δ = 2.50 ppm). ^{1}H-DOSY were recorded on the previously described spectrometer and diffusion coefficients are reported in cm^2 sec^{-1}, obtained using MestReNova 6.02 software Bayesian Method. Diffusion coefficients were calculated from intensity of pyrrole (~3.8 ppm) signal of L-Pro side chain using a mono-exponential decay equation, which is a simplified version of the Stejskal-Tanner function.

Size Exclusion Chromatography. SEC analysis of polyproline (PPro) homopolymers was performed on an Agilent Technologies Infinity 1260 MDS instrument equipped with a differential refractive index (DRI), light scattering (LS) and viscometry (VS) detectors. The column set used were PL Aquagel-OH MIXED-M column. The mobile phase used was a H_2O:ACN mixture (80:20) + 0.1 M $NaNO_3$. Column oven and detector temperatures were regulated to 40°C, at flow rate 1 mL/min. Poly(ethylene oxide) standards (Agilent EasyVials) were used for calibration between 100-500,000 g.mol^{-1}. Analyte samples were filtered through a hydrophilic GVWP membrane with 0.22 μm pore size before injection. Number average molecular weights (M_n), weight average molecular weights (M_w) and dispersities ($Đ_M = M_w/M_n$) were determined by conventional calibration and universal calibration using Agilent GPC/SEC software.

SEC analysis of polysarcosine homopolymers was carried out using a PSS SECurity GPC system equipped with a PFG 7 µm 8 x 50 mm pre-column, a PSS 100 Å, 7 µm 8 x 300 mm and a PSS 1000 Å, 7µm 8 × 300 mm column in series and a differential refractive index (RI) detector at a flow rate of 1.0 mL.min^{-1} in 1,1,1,3,3,3-hexafluoro-2-propanol (HFiP). The system was calibrated against Agilent Easi-Vial linear poly (methyl methacrylate) (PMMA) standards and analysed by PSS winGPCUniChrom. All GPC samples were prepared using a concentration of 2 mg·mL^{-1}, and were filtered through a 0.2 µm millipore filter prior to injection.

FTIR Spectroscopy. Fourier Transform-Infrared (FTIR) spectroscopy measurements were carried out using an A Perkin-Elmer Spectrum 100 spectrometer, in the range of 650 to 4000 cm^{-1} and analyzed using OMNIC™ software.

Turbidimetry. Turbidimetric analysis was performed an Agilent Cary 60 UV-vis spectrophotometer equipped with a Peltier heating and cooling system. Aqueous solutions of PPro polymer samples were prepared at 5 mg.mL^{-1} in PBS with changes in transmittance monitored at λ = 700 nm by heating each sample from 20 °C to 85 °C at a rate of 5 °C.min^{-1}. The inflection point of each thermal phase transition curve was used to determine the lower critical solution temperature (LCST) in each case.

Dynamic Light Scattering. Hydrodynamic diameters (D_h) and size distributions of PPro polymer samples were determined by dynamic light scattering (DLS) using a Malvern Zetasizer Nano ZS with a 4 mW He-Ne 633 nm laser module operating at 25 ^{o}C. Measurements were carried out at an angle of 173° (back scattering), and results were analyzed using Malvern DTS 7.03 software. All determinations were repeated 5 times with at least 10 measurements recorded for each run.

Transmission Electron Microscopy. Dry-state TEM imaging was performed on either a JEOL JEM-2100 or a JEOL JEM-2100Plus microscope operating at an acceleration voltage of 200 kV. All dry-state samples were diluted with MilliQ water and then deposited onto formvar-coated copper grids. After roughly 1 min, excess sample was blotted from the grid and the grid was stained with an aqueous 1 wt% uranyl acetate (UA) solution for 1 min prior to blotting, drying and microscopic analysis.

Cryogenic transmission electron microscopy (cryo-TEM) imaging was performed on a JEOL JEM-2100Plus microscope operating at an acceleration voltage of 200 kV. 5 uL of sample at 0.5% w/w solids content in MilliQ water was plunge-frozen into 30%propane/ethane on a freshly glow-discharged 200 mesh copper grid with lacey carbon coating (EM Resolutions, UK) using a Leica GP2, blotting for 5s at 4 degrees and 95% humidity. The grid was imaged on a JEOL 2100+ with Gatan OneView Camera.

Circular Dichroism. Circular dichroism (CD) analysis of poly(proline) homopolymers was performed on a Jasco J-1500 CD spectrometer, featuring a 150 W air-cooled Xe lamp and a Jasco Peltier PTC-423S/15 temperature controlling system. The sample was prepared at 0.1 mg/mL concentration, in 10 mM sodium phosphate buffer (pH = 7.4) and contained in a 1mm pathlength quartz cuvette (Hellma, USA) with a spectral bandwidth of 1.0 nm. All spectra obtained were the mean of six independent scans post calibration with sodium phosphate buffer. Data collected were analyzed and processed with the online tool "DICHROWEB".

Experimental Procedures

Splat Ice Recrystallization Inhibition Assay. Splat cooling assays were performed as previously described by Tomczak *et al.*[1] Briefly, a 10 µL sample was dropped 1.40 m onto a chilled glass coverslip, resting on a thin aluminium block cooled to -78 °C placed on dry ice. Upon hitting the coverslip, a wafer with diameter of approximately 10 mm and thickness 10 µm was formed instantaneously. The glass coverslip was transferred onto the Linkam cryostage and held at -8 °C using liquid nitrogen for 30 minutes. Photographs were obtained using an Olympus CX 41 microscope with a UIS-2 20x/0.45/∞/0-2/FN22 lens and crossed polarisers (Olympus Ltd), equipped with a Canon DSLR 500D digital camera. Images were taken of the initial wafer (to ensure that a polycrystalline sample had been obtained) and again after 30 minutes. Image processing was conducted using ImageJ. In brief, the number of ice crystals in the field of view was measured for each photograph. The average (mean) of these three measurements was then calculated to find the mean grain area (MGS). The average value and error were compared to that of PBS solution, as appropriate, as a negative control.

Sucrose Sandwich Ice Recrystallisation Inhibition Assay. Sucrose sandwich IRI assays were performed as described by Smallwood *et al.*[2,3] Briefly, 2 µL of $PPro_{50K}$ (10 $mg.mL^{-1}$) containing

45wt % sucrose was sandwiched between two circular 14 mm glass cover slips, and the edges were sealed with grease. The coverslips were pressed together in order to produce a liquid film about 10–20 μm thick. Samples were cooled at a rate of 20 °C.min^{-1} to a final temperature of −50 °C to induce the formation of polycrystalline ice and held at −50 °C for 2 min using a Linkam Biological Cryostage BCS196 with T95-Linkpad system controller equipped with a LNP95-Liquid nitrogen cooling pump and liquid nitrogen as the coolant (Linkam Scientific Instruments UK). The temperature was then elevated to −8 °C at 10 °C.min^{-1} and held for 30 minutes. During this time, images were recorded every 10 mins using an Olympus CX41 microscope equipped with a UIS-2 20x/0.45/∞/0−2/FN22 lens (Olympus Ltd.) and a Canon EOS 500D SLR digital.Image processing was conducted using ImageJ.

Modified Sucrose Sandwich Ice Shaping Assay. Briefly, 1 mg.mL^{-1} of poly(proline) samples were dispersed in 45 wt % sucrose solution and sandwiched between two glass coverslips and sealed with immersion oil. Samples were cooled to −50 °C on a Linkam Biological Cryostage BCS196 with T95-Linkpad system controller equipped with a LNP95-Liquid nitrogen cooling pump, using liquid nitrogen as the coolant (Linkam Scientific Instruments UK). The temperature was then increased to −8 °C and held for 1 h to anneal. The samples were then heated at 0.5 °C.min^{-1} until few ice crystals remained and then cooled at 0.05 °C.min^{-1} and the shape of ice crystals observed. Micrographs were obtained every 0.1 °C using an Olympus CX41 microscope equipped with a UIS-2 20x/0.45/∞/0−2/FN22 lens (Olympus Ltd.) and a Canon EOS 500D SLR digital. Image processing was conducted using ImageJ.

Ice Shaping using Nanoliter Osmometer. An Otago nanoliter osmometer (Otago Osmometers, Dunedin, New Zealand) was used to measure polymer ice shaping. Briefly, 20 nL droplets of 2 mg.mL^{-1} samples were suspended in type B immersion oil (Type B, cargille immersion oil) on a 6-well cooling plate using a microcapillary system.[2,3] The samples were rapidly frozen by cooling the osmometer to ~ −40 °C. A rapid temperature increase was then conducted until the melting point was reached, and then the temperature was gradually increased (0.01 °C.min^{-1}) until only one ice crystal remained. Just before the ice crystal melted, the temperature was decreased until a discernible growth of the ice crystal was observed. It was observed and photographed with an Olympus CX41 microscope equipped with a UIS 20x/0.45/*/0-2/FN22 lens (Olympus Ltd.) and a Canon EOS 1200D digital SLR camera.

Synthetic Procedures

Synthesis of NCA Proline

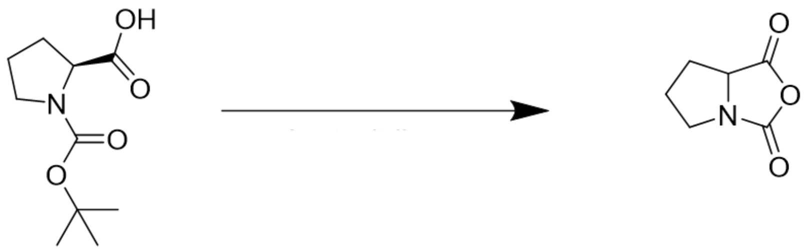

Based on previous method[1]. Boc-L-Proline (5g, 23.23mmol) and epichlrohydrin (8.6g, 92.92mmol) were dissolved in acetonitrile (50mL) at 0 °C and triphosgene (3.45g, 11.6mmol) was added in one portion. The reaction proceeded open to air at 0 °C for 2.5hrs until no solids remained, the solution was filtered and reduced in vacuo at 40 °C for 30 minutes. The flask of the remaining solution was filled with N_2 and using a cannula then precipitated into a large excess of hexane and stored overnight at -18 °C. The resulting solution was reduced down to 1/3rd volume in vacuo under vigorous stirring. The crude NCA oil was dissolved in ethyl acetate and reprecipitated into hexane twice to afford a solid. The NCA was dried overnight over P_2O_5 to afford a white powder (2.4g, yield 85%). ^{1}H NMR (400MHz, $CDCl_3$, 293K) δ 4.33 (m, 1H), 3.79 (dt, J = 11.4, 7.6 Hz, 1H), 3.33 (ddd, J = 11.5, 8.5, 4.9 Hz, 1H), 2.32 (m, 1H), 2.17 (m, 2H), 1.95 (m, 1H). ^{13}C NMR (101 MHz, $CDCl_3$) δ 168.9, 155.0, 63.17, 56.7, 27.26 & 27.0

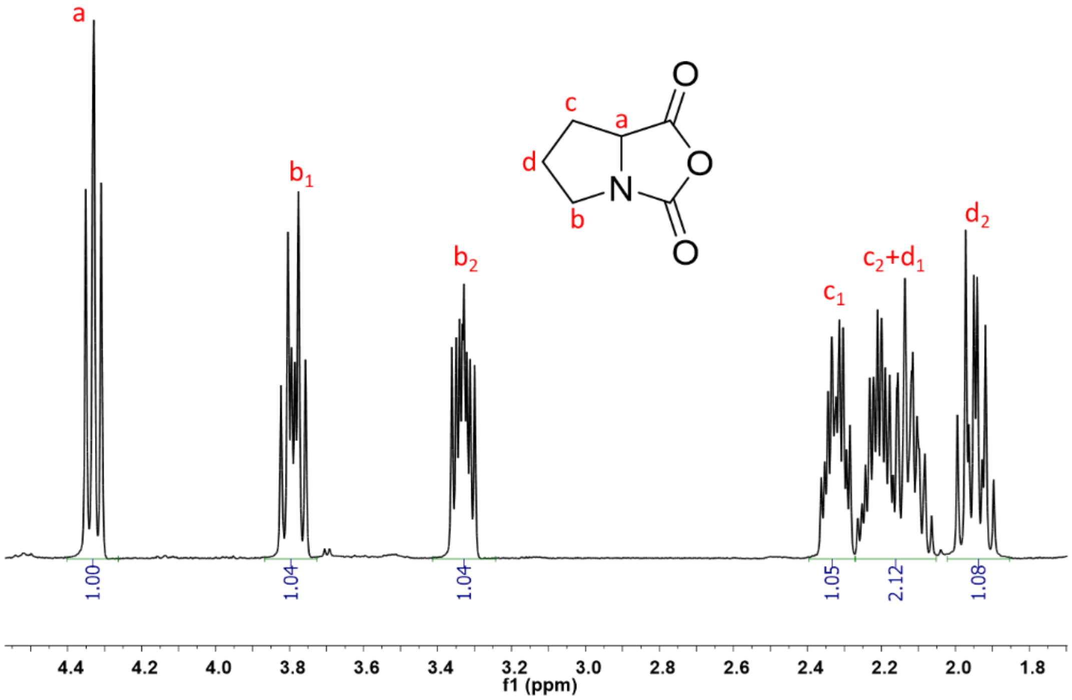

Figure S1. ^{1}H NMR spectra of L-Pro NCA in $CDCl_3$

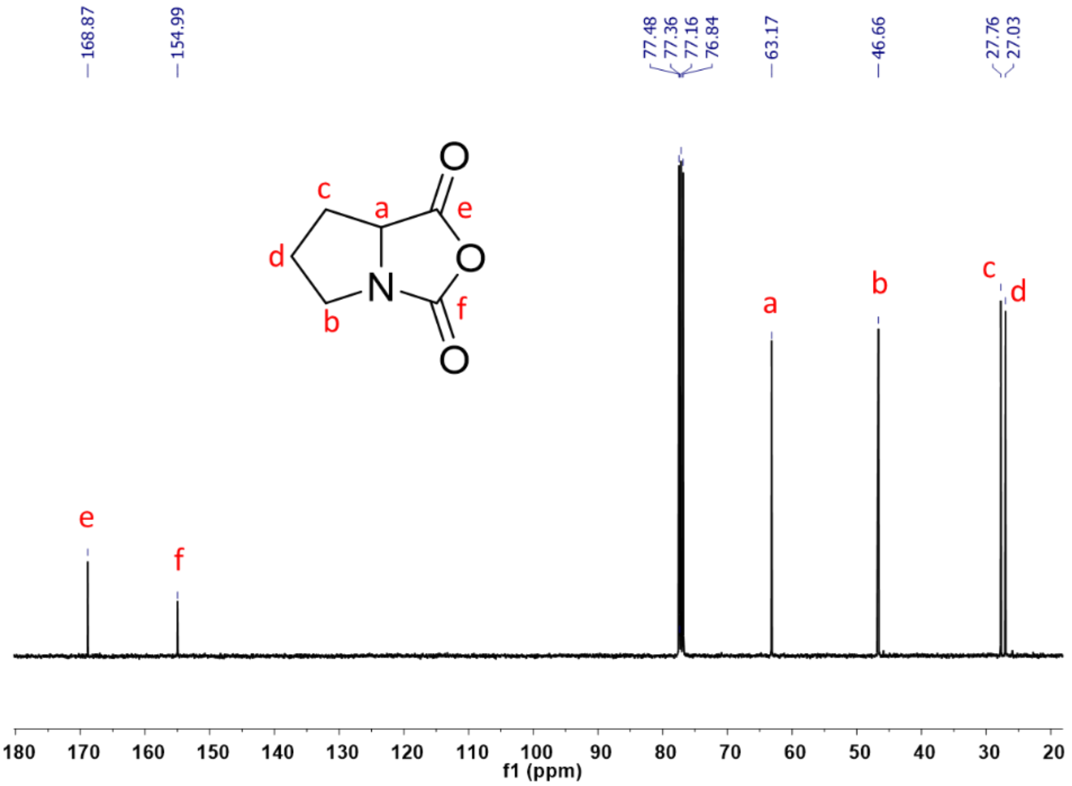

Figure S2. ^{13}C NMR spectra of L-Pro NCA in $CDCl_3$

P (Proline) Synthesis

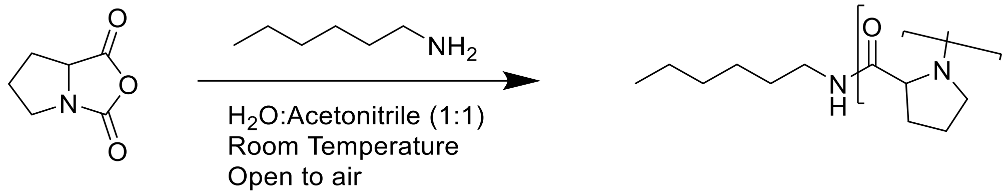

<u>2k, 5k and 10k Targeted M_w</u>

NCA Proline was dissolved in an acetonitrile/H_2O (1:1 v/v) at a concentration of 50mg mL^{-1} in a small vial open to air. Hexylamine was added from a stock solution acetonitrile/H_2O (1:1 v/v) directly in one portion and the reaction proceeded as gas bubbles were evident.[2] Once the NCA peaks at 1823 and 1764 cm^{-1} had disappeared the reaction was quenched by addition of excess of H_2O. The solution was dialysed (3.5kDa MW cut off) for 3 days and lyophilised.

<u>20k and 50k Targeted M_w</u>

The previous procedure was adapted so that NCA Proline was added portion wise in 2 and 5 for the 20kDa and 50kDa targeted Mw respectively. NCA Proline (103 equiv) was dissolved in an acetonitrile/H_2O mixture (1:1 v/v) at a concentration of 50mg mL^{-1} in a small vial open to air. Hexylamine (1 equiv) was added from a stock solution acetonitrile/H_2O (1:1 v/v) directly in one portion and the reaction proceeded as gas bubbles were evident. Upon full monomer consumption, as monitored by FTIR by the disappearance of NCA peaks at 1823 and 1764 cm^{-1}, a subsequent portion of NCA Proline was added. This addition procedure was repeated according to the number of portions required as dictated by the targeted Mw.

^{1}H NMR (400MHz, D_2O, 293K) δ 4.70 (1H), 3.68 (2H), 2.30 (1H), 2.03-1.87 (3H). ^{13}C NMR (101MHz, D_2O) δ 171.6, 58.51, 47.55, 27.86, 24.50.

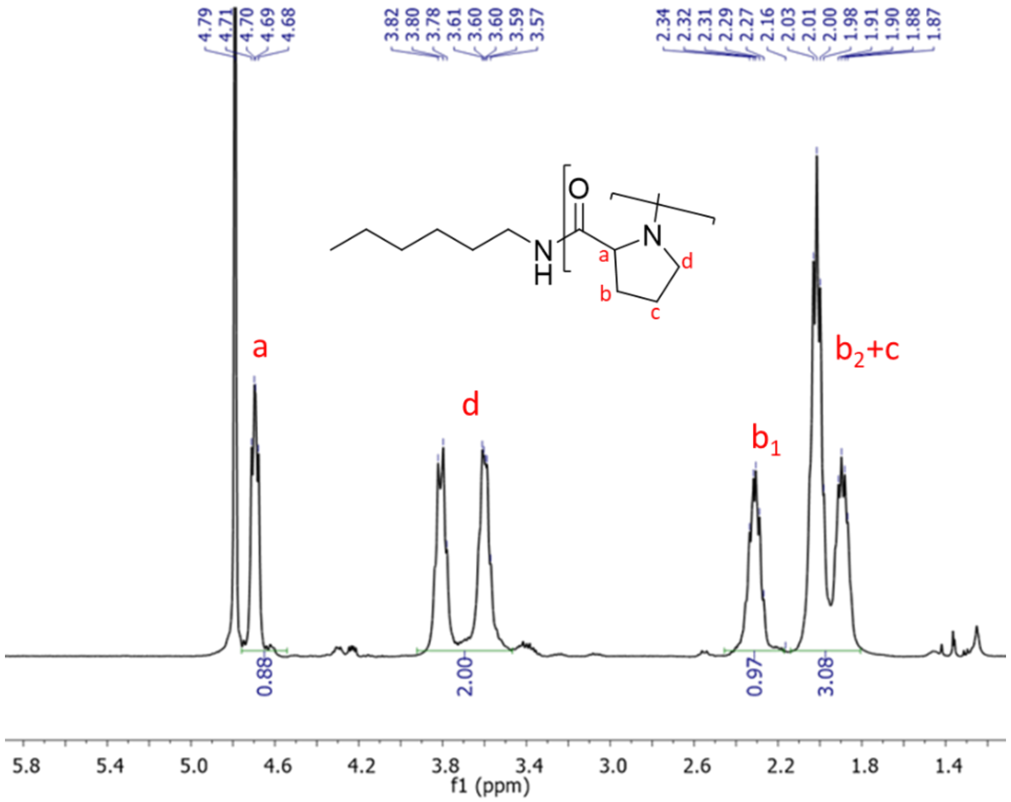

Figure S3. ^{1}H NMR spectra in D_2O of P(L-Pro) with targeted 2k Da M_w.

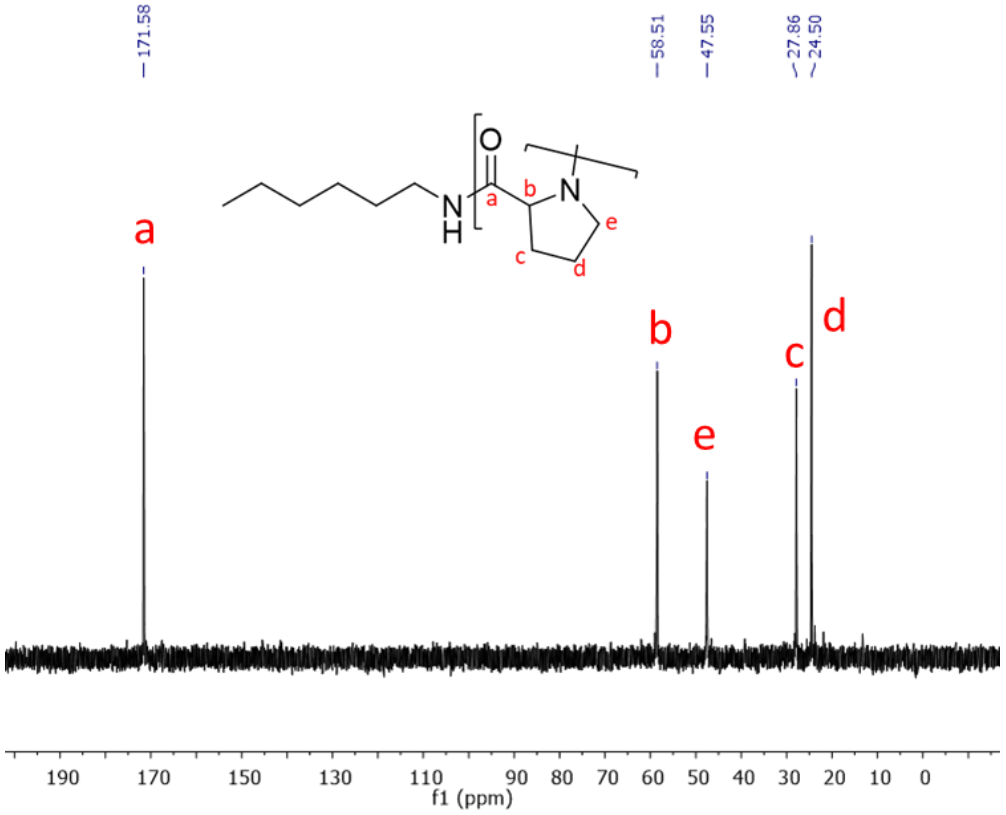

Figure S4. ^{13}C NMR spectra in D_2O of P(L-Pro) with targeted 2k Da M_w.

Sarcosine NCA Synthesis

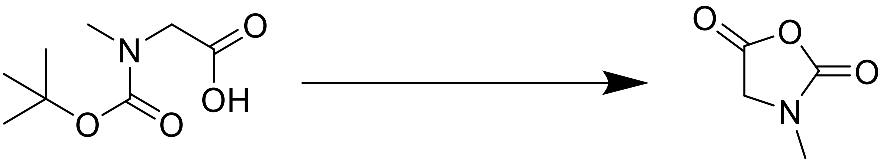

Triphosgene (7.16 g, 24.13 mmol) and epichlorohydrin (8.93 g, 96.52 mmol) were dissolved in 100 mL THF. Pre-dried and ground sarcosine powder (5 g, 56.12 mmol) was then added directly to the solution. The reaction mixture was refluxed until all solids disappeared (3-4 h), leaving a clear solution. The solution was then cooled, bubbled with N_2, filtered, and reduced to 2/3 volume under reduced pressure. It was then precipitated by addition of 120 mL hexane and stored overnight at -18 °C yielding a mixture of brown crystals and a brown oil. The crystals were washed with hexane and dried. They were then redissolved in ethyl acetate (80 mL), precipitated by addition of 240 mL hexane and stored overnight at -18 °C, yielding large white crystals. This process was completed 2 times. For the oil, ethyl acetate was added (50 mL) it was precipitated into excess hexane (240 mL) thrice yielding long shiny crystals (yield: 4.35g, 67%). ^{1}H NMR (400MHz, d_6-DMSO, 293K) δ 4.21 (s, 2H), 2.85 (s, 3H). ^{13}C NMR (101 MHz, d_6-DMSO) δ 167.87, 124.67, 51.62, 30.29.

Polysarcosine (P(L-Sar)) Synthesis

NCA Sar was dissolved in DMF at a concentration of 50mg mL^{-1} and fully dissolved. Hexylamine was added directly in one portion and the reaction proceeded as gas bubbles were evident. The reaction continued overnight and the FTIR spectrum was taken to confirm the disappearance of anhydride peaks at 1823 and 1764 cm^{-1} before quenching by precipitation into diethyl ether. The solid was collected by centrifugation, resuspended in chloroform and precipitated into diethyl ether (x2). The resulting white solid was analysed by ^{1}H NMR and SEC. Various Mw's were targeted by varying the mass of Hexylamine added, table S1.

Table S1. P(L-Sar)s synthesised

**Polymer**	**[M]:[I]**	$M_{n,theo}$ (g.mol^{-1})	$M_{n,NMR}$ (g.mol^{-1})	$M_{n,SEC}$ (g.mol^{-1})	$Đ_M$ (-)
P(L-Sar)$_{2k}$	28	2,000	1,880	14,430	1.03
P(L-Sar)$_{5k}$	70	5,000	4,970	20,950	1.03
P(L-Sar)$_{10k}$	141	10,000	10,100	25,210	1.05
P(L-Sar)$_{20k}$	282	20,000	18,140	27,730	1.07

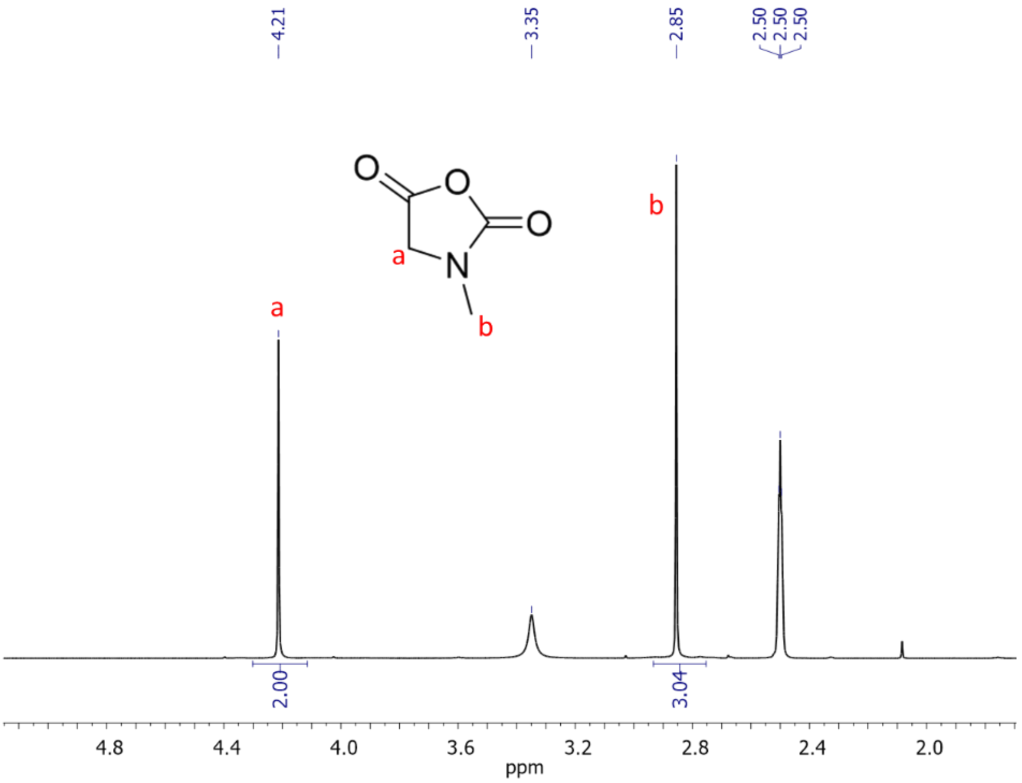

Figure S5. ^{1}H NMR spectra of L-Sar NCA in d_6-DMSO.

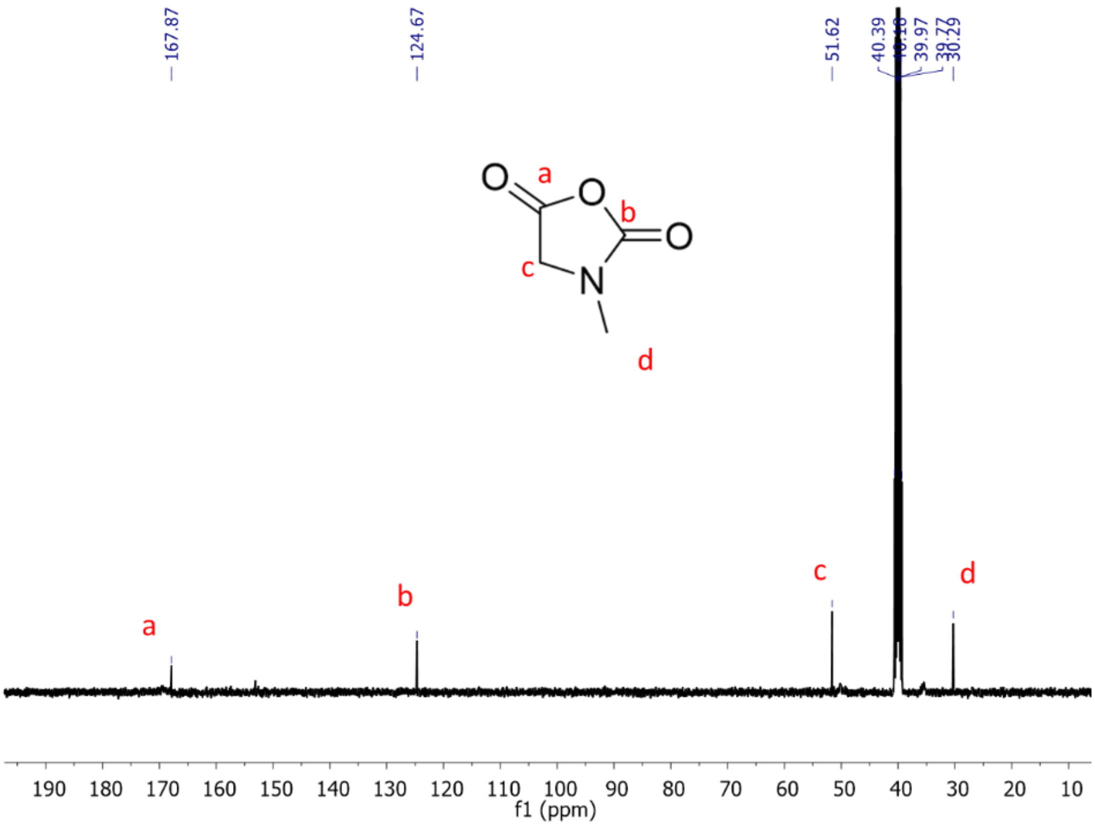

Figure S6. ^{13}C NMR spectra of L-Sar NCA in d_6-DMSO.

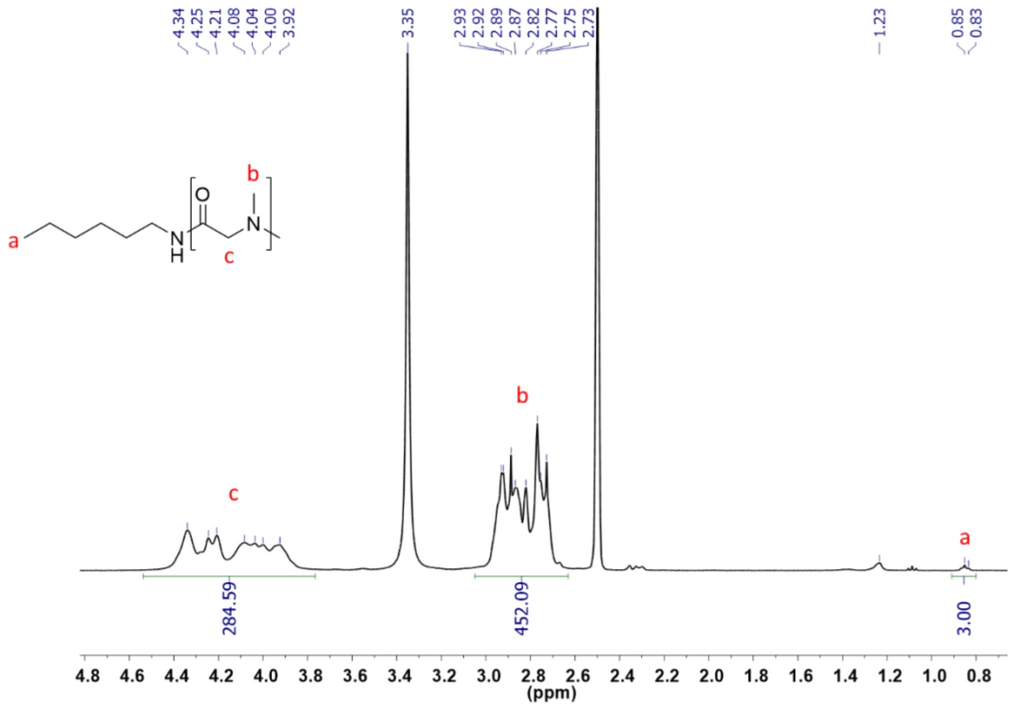

Figure S7. ^{1}H NMR spectra in d_6-DMSO of P(L-Sar) with targeted 10 kDa M_w.

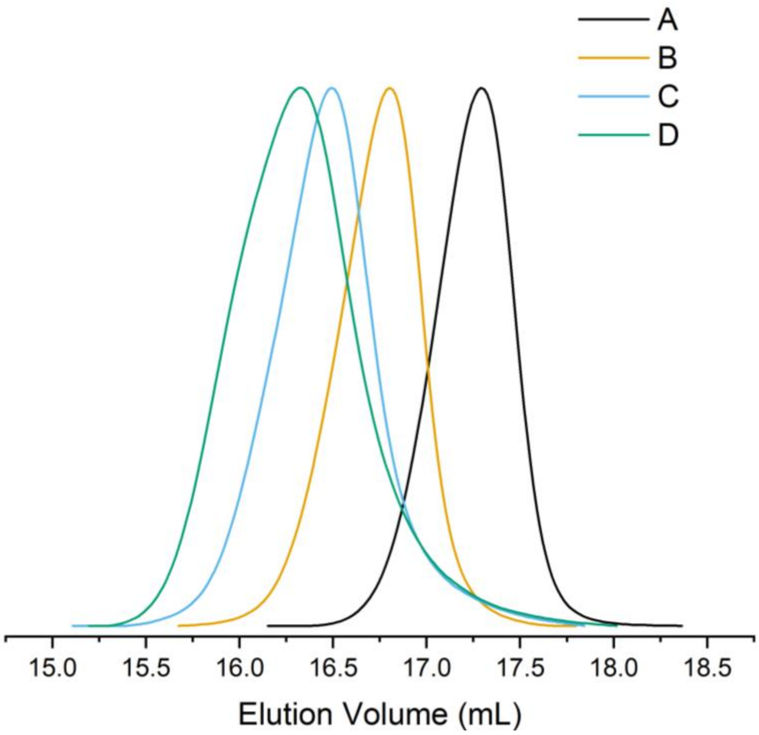

Figure S8. HFiP SEC traces for P(L-Sar) targeted M_w of 2k (A, black), 5k (B, yellow), 10k (C, blue) and 20k Da (D, green).

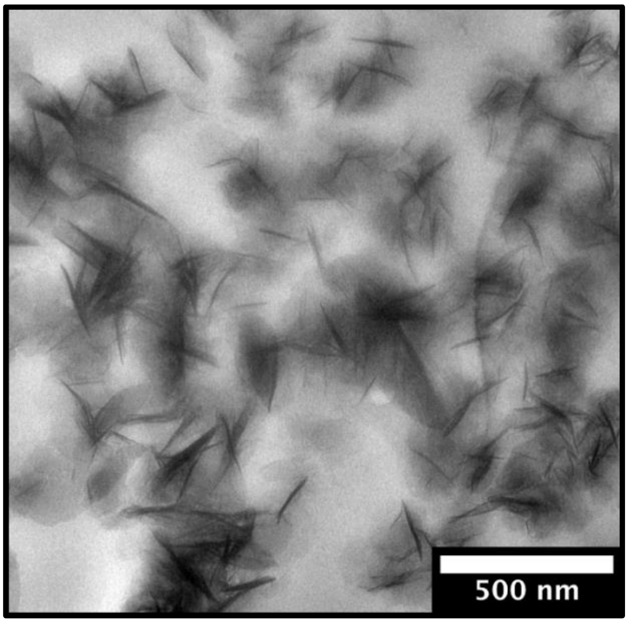

Figure S9. Representative dry-state TEM image of $PPro_{50k}$

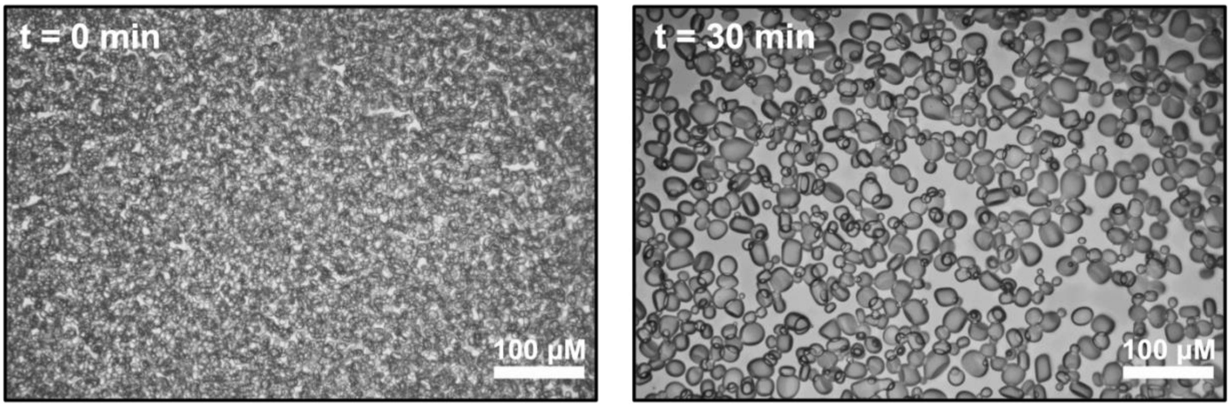

Figure S10. Example cryomicrographs in a sucrose-sandwich (45 wt % sucrose) assay at – 20 °C containing no additives.

References

1. Z.-Y. Tian, Z. Zhang, S. Wang and H. Lu, *Nature Communications*, 2021, **12**, 5810.
2. Y. Hu, Z.-Y. Tian, W. Xiong, D. Wang, R. Zhao, Y. Xie, Y.-Q. Song, J. Zhu and H. Lu, *National Science Review*, 2022, DOI: 10.1093/nsr/nwac033, nwac033.
